# International Clinics

**Published:** 1892-03

**Authors:** 


*International Clinics*. Vol. II. Pp. 356. Edinburgh: YOUNG J. PENTLAND. 1891.

The editors have again presented us with a very useful and instructive series of clinical essays. The subjects treated of are representative of all the great divisions of the healing art. The general level of the contributions is of a high order of excellence, as we should expect from the well-known names of the contributors. From the nature of the book it is of course not to be expected that all the lectures should be equally good, but we can safely affirm that there is not one which will not repay perusal. Their general aim is above all practical, and they must prove of great value to the practitioner by placing him *au courant* of the views held on the diseases discussed by those who have attained to eminence in studying them.

